# Supplementary figures and images for: Bioinformatic Assessment and Expression Profiles of the AP2/ERF Superfamily in the Melastoma dodecandrum Genome
Source: Int J Mol Sci. 2023 Nov 15;24(22):16362. doi: 10.3390/ijms242216362 (PMC10671166; doi:10.3390/ijms242216362)

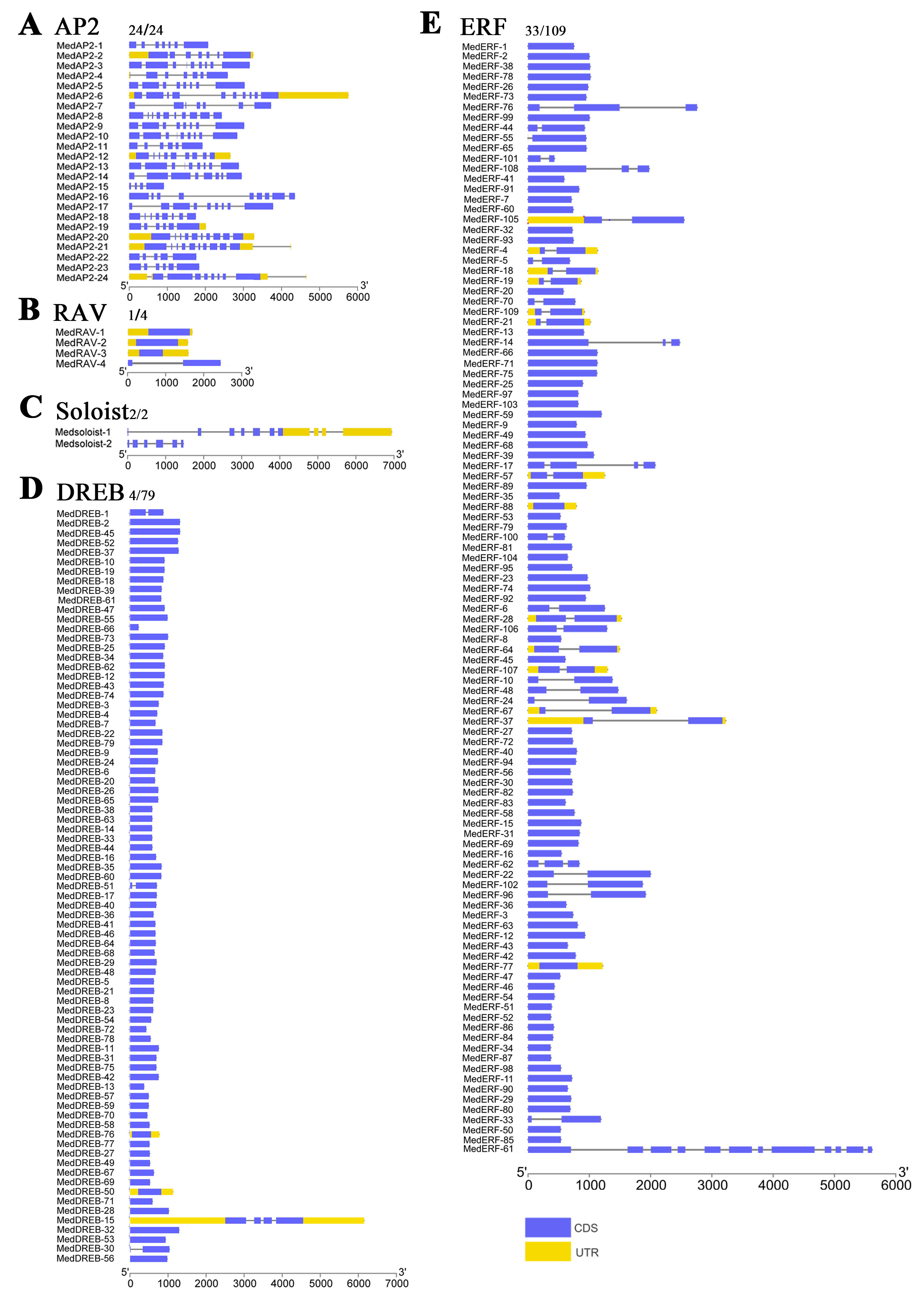

Supplement: Supplementary file 1 [file ijms-24-16362-s001.zip › Supplementary Fugure S1 gene structure.jpg]
